# Naive and Stem Cell Memory T Cell Subset Recovery Reveals Opposing Reconstitution Patterns in CD4 and CD8 T Cells in Chronic Graft vs. Host Disease

**DOI:** 10.3389/fimmu.2019.00334

**Published:** 2019-03-06

**Authors:** Maria V. Soares, Rita I. Azevedo, Inês A. Ferreira, Sara Bucar, Ana C. Ribeiro, Ana Vieira, Paulo N. G. Pereira, Ruy M. Ribeiro, Dario Ligeiro, Ana C. Alho, António S. Soares, Nádia Camacho, Carlos Martins, Fernanda Lourenço, Raul Moreno, Jerome Ritz, João F. Lacerda

**Affiliations:** ^1^JLacerda Lab, Hematology and Transplantation Immunology, Instituto de Medicina Molecular, Faculdade de Medicina da Universidade de Lisboa, Lisbon, Portugal; ^2^Unidade de Citometria de Fluxo, Instituto de Medicina Molecular, Faculdade de Medicina da Universidade de Lisboa, Lisbon, Portugal; ^3^Laboratório de Biomatemática, Faculdade de Medicina da Universidade de Lisboa, Lisbon, Portugal; ^4^Lisbon Centre for Blood and Transplantation, Instituto Português do Sangue e Transplantação, IP, Lisbon, Portugal; ^5^Serviço de Hematologia e Transplantação de Medula, Hospital de Santa Maria, Centro Hospitalar Lisboa Norte, Lisbon, Portugal; ^6^Department of Medical Oncology, Dana-Farber Cancer Institute, Harvard Medical School, Boston, MA, United States

**Keywords:** chronic graft vs. host disease, hematopoietic stem cell transplantation, T lymphocyte, stem cell memory, Naive T cell, immune reconstitution

## Abstract

The success of allogeneic hematopoietic stem cell transplantation (allo-HSCT) in the treatment of hematological malignancies remains hampered by life-threatening chronic graft vs. host disease (cGVHD). Although multifactorial in nature, cGVHD has been associated with imbalances between effector and regulatory T cells (Treg). To further elucidate this issue, we performed a prospective analysis of patients undergoing unrelated donor allo-HSCT after a reduced intensity conditioning (RIC) regimen containing anti-thymocyte globulin (ATG) and the same GVHD prophylaxis, at a single institution. We studied T cell subset homeostasis over a 24-month follow-up after HSCT in a comparative analysis of patients with and without cGVHD. We also quantified naive and memory T cell subsets, proliferation and expression of the apoptosis-related proteins Bcl-2 and CD95. Finally, we assessed thymic function by T cell receptor excision circle (TREC) quantification and T cell receptor (TCR) diversity by TCRVβ spectratyping. While the total number of conventional CD4 (Tcon) and CD8 T cells was similar between patient groups, Treg were decreased in cGVHD patients. Interestingly, we also observed divergent patterns of Naive and Stem Cell Memory (SCM) subset recovery in Treg and Tcon compared to CD8. Patients with cGVHD showed impaired recovery of Naive and SCM Tcon and Treg, but significantly increased frequencies and absolute numbers of Naive and SCM were observed in the CD8 pool. Markedly increased EMRA CD8 T cells were also noted in cGVHD. Taken together, these results suggest that Naive, SCM and EMRA CD8 play a role in the emergence of cGHVD. Reduced Naive and recent thymic emigrant Tcon and Treg in cGVHD was likely due to impaired thymic output, as it was accompanied by decreased CD4 TREC and TCR diversity. On the other hand, CD8 TCR diversity was similar between patient groups. Furthermore, no correlation was observed between CD8 TREC content and Naive CD8 numbers, suggesting limited thymic production of Naive CD8 T cells in patients after transplant, especially in those developing cGVHD. The mechanisms behind the opposing patterns of CD4 and CD8 subset cell recovery in cGVHD remain elusive, but may be linked to thymic damage associated with the conditioning regimen and/or acute GVHD.

## Introduction

Despite the recent advances with patient-tailored therapies, allogeneic hematopoietic stem cell transplantation (allo-HSCT) has been increasingly used in the USA and in Europe for the treatment of hematological malignancies (1, 2). However, this technique is not without risks and is frequently accompanied by serious complications such as graft vs. host disease (GVHD) and infections, which are major causes of morbidity and mortality post-transplant (3). GVHD results from the recognition of patient tissues by donor-derived effector cells. Acute GVHD (aGVHD) typically occurs early after transplant, has markedly inflammatory manifestations and is thought to be primarily mediated by mature T lymphocytes infused with the graft (4). On the other hand, chronic GVHD (cGVHD) usually occurs later after transplant and resembles an auto-immune disease (5), affecting specific target organs, primarily the eyes, mouth, gastrointestinal tract, liver, skin, lungs, musculoskeletal, and genitourinary systems (6). Chronic GVHD has a complex pathophysiology. Thymic damage resulting both from the conditioning regimen and acute GVHD likely has an impact on normal T cell development (7, 8), and self-reactive T cells are believed to play a pivotal role in the development of cGVHD. Moreover, there appears to be a deficit in the regulatory T cell pool, contributing to the loss of immunologic tolerance post-transplant. A pivotal role for autoreactive B cells and the production of self-reactive antibodies has also been clearly associated to cGVHD pathogenesis, whereby self-reactive B lymphocytes are activated due to increased levels of BAFF (9, 10). In the end, excessive macrophage activation leads to fibroblast proliferation and collagen deposition, which is a hallmark of cGVHD (11).

The imbalances in regulatory (Treg) and effector T cells (12) appear to be central to cGVHD pathogenesis. This has led to clinical trials investigating the effect of low-dose rhIL-2 in patients with cGVHD in order to induce Treg expansion *in vivo* (13, 14). Also with the aim of increasing the Treg pool, we and others are conducting clinical trials of donor Treg infusion in patients with moderate and severe cGVHD (www.tregeneration.eu).

The involvement of donor T cells in the pathophysiology of GVHD led to the development of *ex vivo* (T cell-depleted grafts) and *in vivo* (anti-thymocyte globulin; ATG) T cell depletion approaches that significantly reduce GVHD incidence (5). ATG also delays immune reconstitution post-transplant through the depletion and/or function modification of T, B and NK cells (15). However, ATG does not completely abrogate the emergence of cGVHD (16–18), which attests to the multifactorial nature of this condition. On the other hand, thymic ablation has been shown to prevent cGVHD (8), suggesting a significant role for *de novo* thymic-derived T cells in this pathology.

In this study, we aimed at further investigating the biology of cGVHD and its effects on T cell homeostasis. Given the role that T cell immunity plays in cGVHD, we prospectively evaluated T cell reconstitution and thymic function in a homogenous patient population undergoing allo-HSCT after a reduced intensity conditioning (RIC) regimen containing ATG. We assessed the kinetics of T cell reconstitution after allo-HSCT and performed a comparative analysis of patients developing cGVHD vs. those who did not.

## Materials and Methods

### Patients and Sample Collection

We prospectively monitored 57 patients undergoing allo-HSCT at Hospital de Santa Maria (Centro Hospitalar Universitário Lisboa Norte) from unrelated donors after a RIC regimen containing fludarabine 30 mg/m^2^/day for 5 days (D-8 to D-4), melphalan 70 mg/m^2^/day for 2 days (D-3 and D-2), and ATG (thymoglobulin) 4–6 mg/Kg (total dose) divided in 2–3 days, according to HLA compatibility. GVHD prophylaxis consisted of cyclosporine A (CsA) plus mycophenolate mofetil (MMF) in all patients. CsA and MMF were initiated on D-1 with CsA at 3 mg/kg/day intravenously (*iv*) twice daily and MMF at 2 g/day (*iv* or *per os*). CsA blood levels were monitored to target levels of 200 ng/mL. In the absence of GVHD, immune prophylaxis was tapered and discontinued between months 6 and 9 post-transplant.

Our center has acquired a significant experience with the administration of thymoglobulin in unrelated donor allo-HSCT over the last decade and maintained the same protocol in the present patient cohort.

Seventeen patients were excluded due to early disease relapse or death from either infection or aGVHD in the first 9 months post-transplant. Chronic GVHD diagnosis and staging were performed according to the 2014 NIH criteria (19). Patient and donor characteristics are shown in Table 1. Peripheral blood was collected on months 1, 2, 3, 6, 9, 12, 18, and 24 after HSCT, and from five healthy controls (HC). Up to 2 weeks of variance in sample collection was allowed for patient sample collection.

**Table 1:** Patient/donor characteristics.

	**cGVHD**	**No cGVHD**
Patients	18	22
Gender	6 F; 12 M	12 F; 10 M
Age	51 (30–67)	46 (19–69)
**DIAGNOSIS**		
AML	7	10
ALL	0	4
CLL	1	0
CML	2	2
HL	1	0
NHL	4	3
MDS	2	0
MF	1	0
MM	0	3
CMV Positive	14	18
**DONORS**		
Gender	11 F; 7 M	9 F; 13 M
Age	35 (21–49)	32 (20–57)
Female Donor to Male Patient	6	4
CMV Pos	12	15
**HLA MATCHING**		
10/10	8	9
9/10	9 (3A, 1B, 4C, 1DQB1)	12 (8A, 1B, 2C, 1DQB1)
8/10	1 (DRB1+DQB1)	1 (A+DQB1)
**SOURCE OF GRAFT**		
BM	5	11
PBSC	13	11
aGVHD	17/18	9/22
GI	5	2
GII	6	4
GIII	6	3
GIV	–	–
cGVHD	18	–
Mild	7	–
Intermediate	4	–
Severe	7	–
Day of cGVHD Onset	230 (149–455)	–
Follow Up (days)	682 (286–1,051)	483 (90–840)

Written informed consent was obtained before sample collection in accordance with the Declaration of Helsinki. This study obtained approval from the Ethics Committee of Lisbon Academic Medical Center (ref. 459/13).

### Flow Cytometry

Flow cytometry analysis was performed on fresh whole blood, at pre-determined fixed time-points as indicated above, using the Human Regulatory T Cell Whole Blood Staining Kit (eBioscience), according to the manufacturer's instructions and immediately acquired on a LSR Fortessa (BD Biosciences).

The following monoclonal antibodies were used: Bcl-2 (Bcl-2/100) and CD31 (WM-59) FITC; Ki-67 (20Raj1) and CD95 (DX2) PE; CD3 (OKT3) PerCPCY5.5, CD45RA (HI100) and CD25 (M-A251) PE-CY7; CD4 (RPAT4) APC; CD127 (eBioRDR5) and CD62L (DREG-56) APCeFlour780 and FoxP3 (PCH101) e450 (all from eBioscience, except for CD25 PE-CY7, Becton Dickinson). Daily flow cytometer quality control monitoring was performed using Cytometry Setup and Tracking Beads (Becton Dickinson). Eight peak calibration Rainbow Beads (Becton Dickinson) were used to ensure stable fluorescence measurements throughout the study. Data was analyzed without prior knowledge of cGVHD status, using FlowJo9®. For quantification of Ki-67, Bcl-2, and CD95 within T cell subsets a cutoff of 50 events was used as a valid data point for statistical analysis. Five healthy control samples were stained in the same conditions.

### CD4 and CD8 T Cell Receptor (TCR) Repertoire Analysis

TCR Diversity was evaluated with a TCRβgene (TRB) complementarity-determining region 3 (CDR3) spectratyping assay, performed on patient samples from either early (month 3) or late (month 9 or 12) time-points post-HSCT, as previously described (20). Cryopreserved PBMC from a separate set of 8 healthy controls, 10 patients with, and 10 without cGVHD were thawed, stained for CD3, CD4 and CD8, and FACSorted (BD FACSAria III) into CD4 and CD8 T cells. Total RNA isolated from each cell fraction (AllPrep, Qiagen) was used for first-strand cDNA synthesis primed with an equivolume mixture of random hexamers and oligo (dT) (Invitrogen Superscript III). TCRβ transcripts were amplified with TRBV family specific primers and a common TRCB reverse primer (21, 22). A run-off reaction with a second TRCB FAM-labeled primer was used to extend these products. Each fluorescent TRBV-TRBC fragment was separated using capillary electrophoresis-based DNA automated sequencer. Data were analyzed with GeneMapper (Thermo Fischer Scientific) for size, peak count, and fluorescent intensity determination. Profiles of transcript TRB repertoires were classified based on peak count, distribution shape and relative fluorescence intensity (RFI) of each peak (% RFI = 100 × clonal peak area/total peak area) (23).

### Signal-Joint TCR Excision Circle (sjTREC) Quantification in CD4 and CD8 T Cells

sjTREC sequences were analyzed with a multiplex qPCR assay (24) in CD4 and CD8 T cells using the same samples purified for the diversity studies. sjTREC sequence copy numbers were extrapolated from standard curves obtained by 10-fold serial dilutions of a triple-insert plasmid containing sjTREC and TRAC fragments in a 1:1:1 ratio (kind gift from L. Imberti, Spedali Civili of Brescia, Italy). Detected sjTREC copies were genome normalized with the mean quantity of T cell receptor α-chain sequences. Results were expressed as sjTREC copies per 10^6^ CD4 or CD8 T lymphocytes.

### Statistical Analysis

The patients' clinical data shown in Table 1 was compared using Fisher's exact test and the Mann–Whitney test when comparing continuous data. Data obtained for patients with and without cGVHD were compared at each time-point using the Mann-Whitney test on GraphPad Prism®. To analyze slopes of reconstitution early (up to 6 months) and late (from 6 months onwards) post-HSCT, we used linear mixed effects (LME) models over those two periods. In this approach, the data of all patients over time is analyzed together, with patient as the random effect, while time and cGVHD (yes/no) are covariates. In each case, we tested also for an interaction term of cGVHD with time (i.e., different slopes of reconstitution). Previous aGVHD, gender, and source of graft were further included as covariates. In all cases, statistical significance was assessed at α = 0.05.

## Results

### Patient Characteristics

All patients underwent allo-HSCT from an unrelated donor and received the same conditioning and GVHD prophylaxis regimens, as described in the methods section. Patient and donor characteristics are summarized in Table 1. The most frequent underlying disease within patients included in the analysis was AML (42.5%) and 60% of transplants used PBSC as the source of graft. After a 24-month follow-up post-HSCT, 18 patients developed cGVHD while 22 did not (No cGVHD). Median cGVHD onset day was 230. Patients without cGVHD had a shorter follow-up compared to cGVHD patients. Seventeen out of 18 cGVHD patients had previously developed aGVHD, in contrast to 9 out of 22 in the No cGVHD group. No significant differences were identified when comparing the patient characteristics between groups (Table 1), with the exception of the higher incidence of aGVHD in patients with cGVHD (*p* = 0.0006). Five healthy controls (HC), with a median age of 43 (range 36–45), were also studied.

### Distinct Treg, Tcon, and CD8 Reconstitution Patterns After HSCT

**Figure 1:**
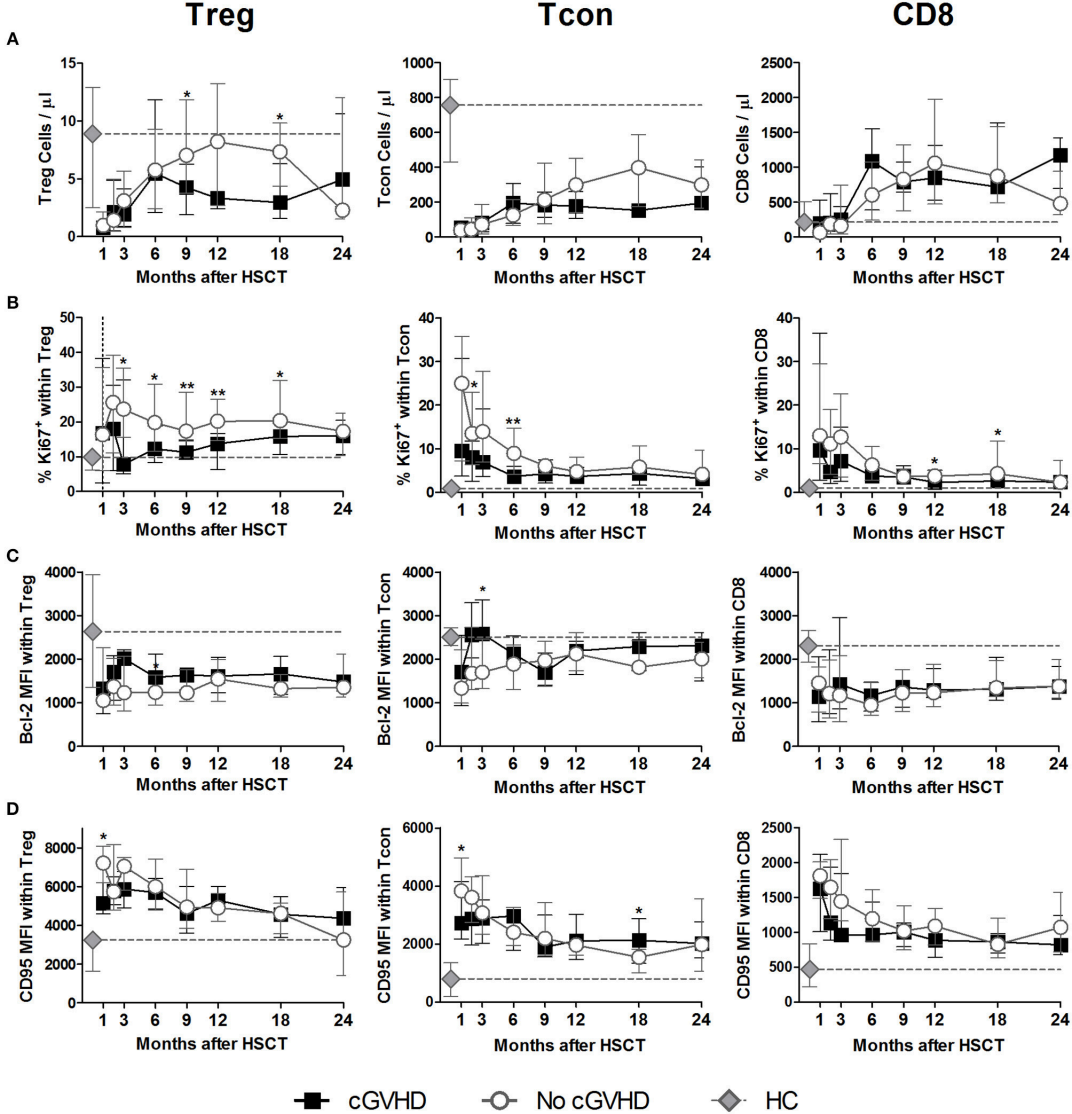
Treg, Tcon and CD8 Homeostasis following HSCT. Patients were divided into No cGVHD (gray lines and open circles) and cGVHD (black lines and squares). Healthy controls (HC) are also shown (gray diamonds and dotted gray lines). **(A)** Absolute counts per microliter for Treg (CD3^+^ CD4^+^ CD25^bright^ Foxp3^+^ CD127^low^), Tcon (CD3^+^ CD4^+^ Foxp3^−^), and CD8 (CD3^+^ CD4^−^) T cells. **(B)** Frequency of Ki-67^+^ cells within CD3^+^ CD4^+^ Foxp3^+^ Treg, Tcon, and CD8 T cells. Median fluorescence intensity (MFI) for Bcl-2 **(C)** and CD95 **(D)** within CD3^+^CD4^+^ Foxp3^+^ Treg, Tcon and CD8 T cells. Symbols represent median values and whiskers represent interquartile range (IQR). Asterisks denote statistically significant differences between patient groups (^*^p = 0.01 to 0.05; ^**^p = 0.001 to 0.01).

Treg numbers were low in both patient groups up to month 6 after HSCT (Figure 1A). From months 9 to 18, Treg were decreased in cGVHD vs. No cGVHD patients. Analysis of *ex vivo* proliferation using intracellular Ki-67 staining revealed significantly decreased proliferation from months 3 to 18 in patients developing cGVHD as compared to No cGVHD, suggesting that reduced Treg numbers in cGVHD may be partly due to reduced homeostatic proliferation (Figure 1B).

Lower Treg numbers in cGVHD were not associated with increased susceptibility to apoptosis as assessed by Bcl-2 and CD95 expression levels, as these proteins were expressed at similar levels in both patient groups (Figures 1C,D). These data suggest that apoptosis mediated by these pathways did not play a major role in the low Treg numbers observed in cGVHD.

We further quantified conventional CD4 T (Tcon; Foxp3^−^ CD4) and CD8 T cell numbers and found no major differences between patients with and without cGVHD (Figure 1A). We did observe significant reductions in Tcon and CD8 proliferation, as assessed by Ki-67 staining, in cGVHD compared to No cGVHD (Figure 1B). On the other hand, no clear trend for altered Bcl-2 or CD95 expression in cGVHD was observed (Figures 1C,D).

### Impaired Naive and SCM Treg Reconstitution in cGVHD

**Figure 2:**
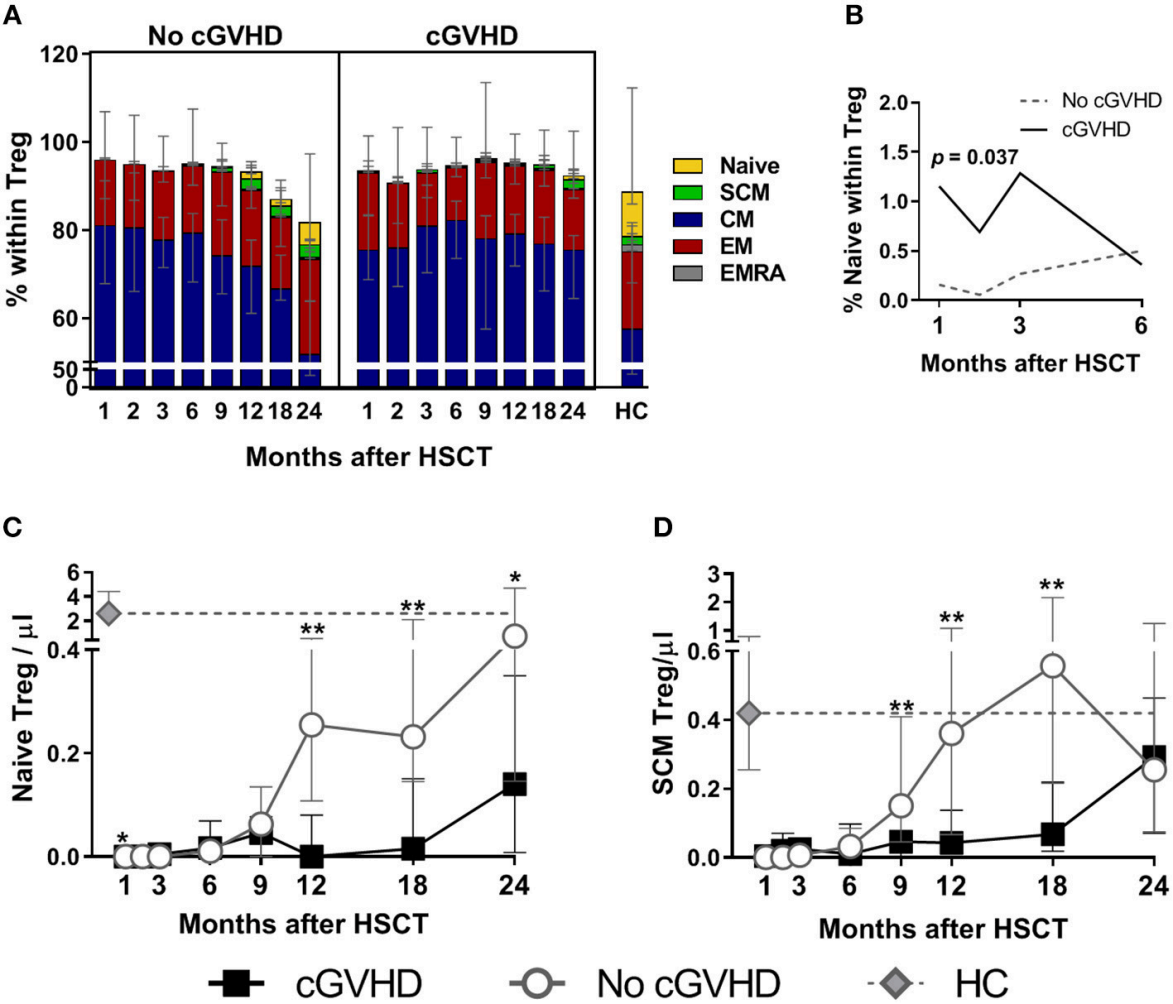
Treg subset reconstitution in cGVHD. Treg were identified as CD4^+^ Foxp3^+^ within a CD3^+^ lymphocyte gate. Naive and memory subsets were identified as shown in Supplementary Figure 1. **(A)** The median percentage for each subset within Treg is shown for patients with and without cGVHD, as well as for HC. **(B)** Naive Treg percentages in the first 6 months after HSCT in patients with cGVHD (black line) and No cGVHD (dotted gray line), illustrating the results of the LME analysis for this subset. Absolute counts of Naive **(C)** and SCM **(D)** Treg in patients without cGVHD (gray lines and open circles), with cGVHD (black lines and squares) and HC (gray diamonds and dotted gray lines). Symbols represent median values and whiskers represent IQR. Asterisks denote statistically significant differences between patient groups (^*^p = 0.01 to 0.05; ^**^p = 0.001 to 0.01).

Quantification of Naive/memory Treg subsets was performed by flow cytometry as described in the methods using CD45RA, CD62L, and CD95 surface markers. We identified Central Memory (CM) cells as CD45RA^−^ CD62L^+^, Effector Memory (EM) as CD45RA^−^ CD62L^−^ and CD45RA-expressing Terminal effectors (EMRA) as CD45RA^+^ CD62L^−^. We further used CD95 to distinguish Naive, CD45RA^+^ CD62L^+^CD95^−^, from the Stem Cell Memory (SCM) subset, CD45RA^+^ CD62L^+^CD95^+^, as previously described (25) (detailed gating strategy illustrated in Supplementary Figure 1A). The percentages of each subset within Treg from patients with and without cGVHD is summarized in Figure 2A. A detailed visualization of the overall data showing percentage and absolute counts for each subset in all time-points for both patient groups and healthy controls, as well as the corresponding significances, is displayed in Supplementary Figure 1B.

The most prevalent subset within Treg was CM, followed by EM, naïve, and SCM, while EMRA were an extremely rare subset (Figure 2A). We observed decreased Naive Treg percentages and absolute counts in cGVHD as compared to No cGVHD throughout the follow-up (Figures 2A,C). The only exception was at Month 1 when Naive Treg frequency and absolute counts were increased in cGVHD vs. No cGVHD patients (Figures 2A,C). Despite this, Naive Treg remained low in patients developing cGVHD while they increased in the absence of cGVHD. This trend, reflecting an inability of Naive Treg to recover in patients developing cGVHD, was confirmed in a LME analysis of the first 6 months post-HSCT. This analysis showed significantly different slopes estimated for the recovery of Naive percentages, whereby Naive Treg percentages only increased in the absence of cGVHD (Figure 2B). From month 9 onwards, Naive Treg percentages and counts were lower in patients developing cGVHD as compared to patients that remained free from cGVHD (Figures 2A,C).

Similar to Naive Treg, the recovery of the SCM Treg subset was impaired in patients developing cGVHD. This was the case for percentages and for absolute counts, which were significantly reduced in cGVHD from month 9 to 18 (Figures 2A,D). The relative proportion of the remaining memory subsets was largely unaltered by cGVHD (Figure 2A). CM and EM Treg absolute counts were either similar or reduced in cGVHD, whereas EMRA Treg were similar or increased, but no sustained statistical significances were noted (refer to Supplementary Figures 1B,C for details).

In summary, cGVHD was associated with impaired Treg recovery, particularly of the Naive and SCM Treg subsets, and lower Treg proliferation as compared to the No cGVHD group.

### Impaired Naive and SCM Tcon Reconstitution in cGVHD

**Figure 3:**
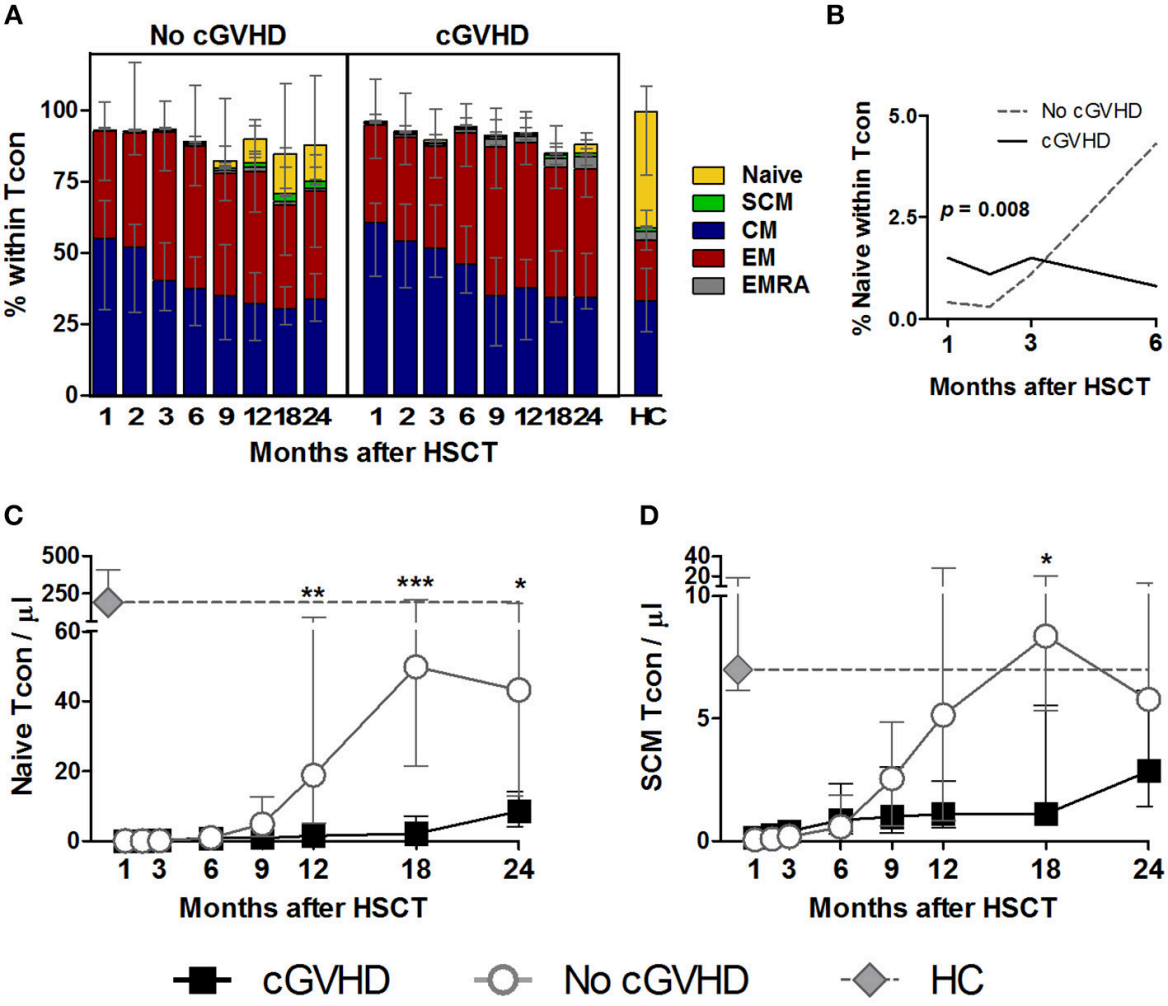
Naive and memory Tcon reconstitution in cGVHD. Tcon were identified as CD4^+^ Foxp3^−^ within a CD3^+^ lymphocyte gate. Naive and memory subsets were identified as shown in Supplementary Figure 1. **(A)** The percentage of each subset within Tcon cells is shown for patients with and without cGVHD, as well as HC. **(B)** Percentage of Naive within Tcon in the first 6 months after HSCT in patients with cGVHD (black line) and No cGVHD (dotted gray line), illustrating the results of the LME analysis for this subset. Naive **(C)** and SCM **(D)** Tcon absolute counts in patients without cGVHD (gray line and circles), patients with cGVHD (black line and squares) and HC (gray diamonds and dotted gray lines). Median values and IQR are shown. Asterisks denote statistically significant differences between patient groups (^*^p = 0.01 to 0.05; ^**^p = 0.001 to 0.01; ^***^p = 0.0001 to 0.001).

Chronic GVHD was associated with a marked impairment in Naive Tcon recovery as compared to patients who did not develop cGVHD. This became apparent from month 9 onwards in terms of both frequency and absolute counts (Figures 3A,C). Although differences between patient groups only reached significance after month 9, the LME analysis revealed that the percentage of Naive Tcon up to month 6 was better represented by a model with significantly different slopes for cGVHD and No cGVHD (*p* = 0.008) (Figure 3B). Hence, during the first 6 months after HSCT, Naive Tcon percentages increased in patients who did not subsequently develop cGVHD, while they remained stable in patients who develop cGVHD. This translated, at later time-points, into significant differences in Naive Tcon frequencies and numbers between patient groups from months 12 to 24 (Figures 3A,C; Supplementary Figures 1B,C). Impaired Naive Tcon recovery in cGVHD was mimicked by decreased SCM Tcon levels, reaching significance at month 18 for SCM Tcon counts (Figures 3A,D).

CM and EM were the most abundant Tcon subsets post-HSCT and were largely similar between patient groups, while EMRA showed a non-significant tendency to be increased in cGVHD (Figure 3A). Percentages and absolute counts for all Tcon subsets are detailed in Supplementary Figures 1B,C.

In summary, we did not observe significant disparities in whole Tcon recovery between patients developing cGVHD and those who did not. However, the composition of the Tcon pool after HSCT reveals an impairment in Naive and SCM subsets in patients who develop cGVHD, similar to what was observed in Treg.

### Increased CD8 Naive, SCM, and EMRA in cGVHD

**Figure 4:**
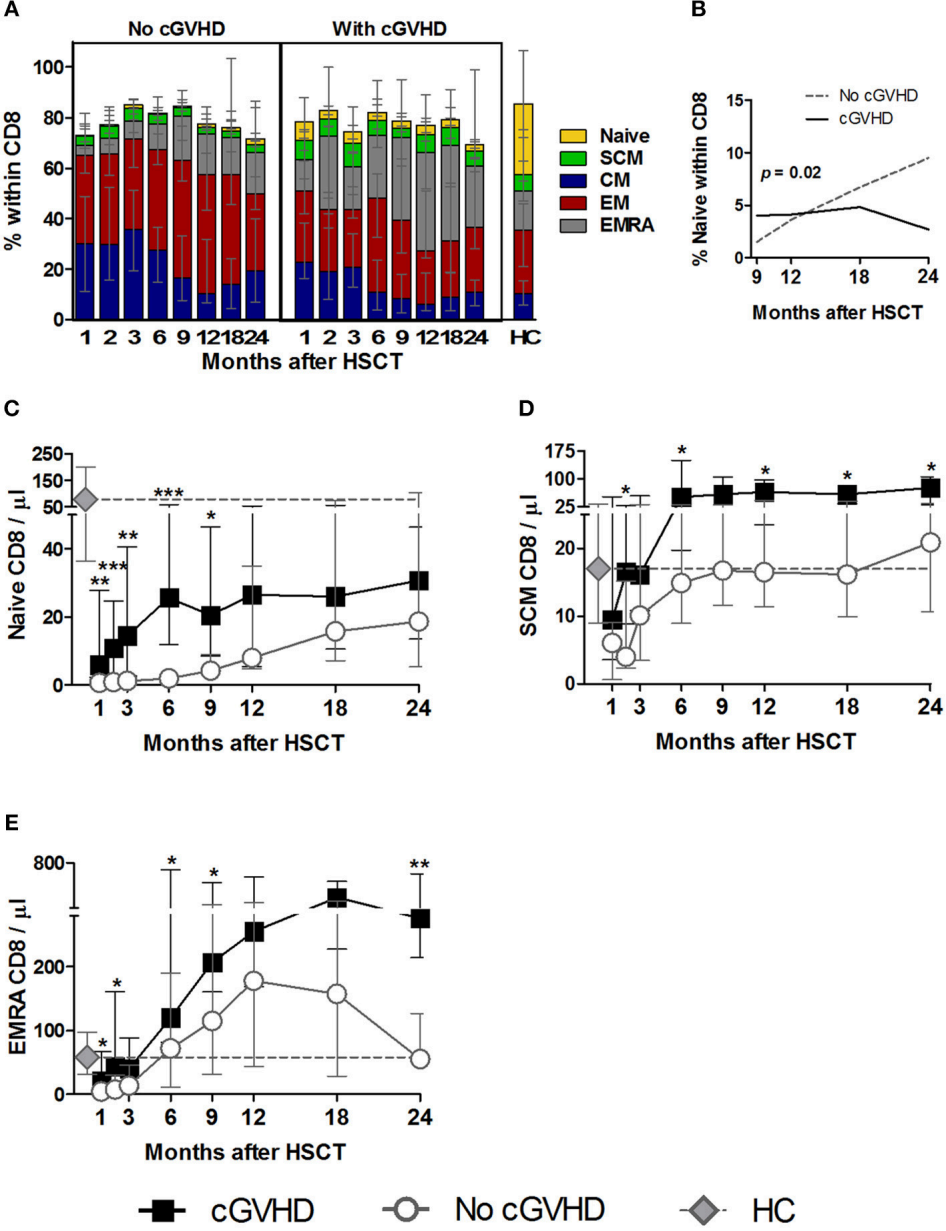
Naive and memory CD8 reconstitution in cGVHD. CD8 T cells were identified as CD3^+^ CD4^−^ lymphocytes. Naive and memory subsets were identified as shown in Supplementary Figure 1. **(A)** Percentage of each subset within CD8 cells for patients with and without cGVHD, as well as HC. **(B)** Naive CD8 percentages from month 9 to 24 after HSCT in patients with cGVHD (black line) and no cGVHD (dotted gray line), illustrating the results of the LME analysis for this subset. Absolute counts of Naive **(C)**, SCM **(D)**, and EMRA **(E)** CD8 cells in patients without cGVHD (gray circles and dotted lines), patients with cGVHD (black squares and lines) and HC (gray diamonds and dotted gray lines). Symbols represent median values and whiskers represent IQR. Asterisks denote statistically significant differences between patient groups (^*^p = 0.01 to 0.05; ^**^p = 0.001 to 0.01; ^***^p = 0.0001 to 0.001).

The reconstitution of Naive and memory subsets within the CD8 T cell pool differed greatly between patient groups (Figure 4) and had a strikingly different pattern to that observed in Tcon and Treg. This was particularly prominent not only for the least abundant Naive and SCM CD8 subsets, but also for the more abundant, terminally differentiated EMRA subset.

Interestingly, we observed significantly increased Naive CD8 percentages and counts in cGVHD vs. No cGVHD during the initial post-transplant period (Figures 4A,C). This statistical significance disappeared from month 12 onwards, as Naive CD8 increased in patients who did not develop cGVHD. Such accelerated Naive CD8 T cell recovery in the absence of cGVHD at later time-points was also revealed by the LME analysis of months 9 to 24 post-HSCT. This analysis showed a significant difference in the slopes for Naive CD8 percentages between the two patient groups (Figure 4B). While in patients with cGVHD the percentage of Naive CD8 stabilized, in the absence of cGVHD the frequency of these cells increased over time.

Interestingly, the SCM CD8 subset was also increased in patients developing cGVHD vs. No cGVHD, starting from the early time points of the follow-up period, both in percentages and absolute counts (Figures 4A,D).

Despite such prominent differences in Naive and SCM subsets, these were always the least abundant populations. Within the most frequent populations the EMRA showed the most striking differences between patient groups. Hence, terminally differentiated EMRA CD8 cells were increased in percentage and counts from the very early post-transplant period in patients developing cGVHD (Figures 4A,E), while the remaining memory subsets, CM and EM, showed an opposite pattern to that observed in Naive, SCM and EMRA, with significant reductions in percentages being noted in patients developing cGVHD (Figure 4A; Supplementary Figures 1B,C).

In summary, CD8 T cell reconstitution in cGVHD was associated with persistently increased Naive, SCM and EMRA subsets. This became apparent very early after HSCT, suggesting a possible involvement of these subsets in disease development.

### The Effects of Acute GVHD on SCM Tcon and CD8

In our patient cohort, all but one of the patients developing cGVHD had a history of previous aGVHD, while within the group of 22 patients who did not develop cGVHD, 9 had developed acute GVHD (aGVHD). In order to clarify the contribution of aGVHD and the associated therapies to differences in T cell reconstitution between patient groups, we tested the effect of adding aGVHD as a covariate in the LME analysis. This analysis showed that during the first 6 months after HSCT, SCM Tcon and CD8 percentages and counts were significantly affected by aGVHD (*p* < 0.01). No impact of aGVHD was observed from month 6 onwards.

To complement this analysis, we stratified the No cGVHD group into acute only (Ac GVHD, *n* = 13) and no GVHD at all (No GVHD, *n* = 9). The only patient in our cohort who did not have aGVHD prior to cGVHD was excluded from this analysis. Hence, the chronic GVHD group consisted of patients with acute and chronic GVHD (Ac & Ch GVHD, *n* = 17). Month 24 was excluded from this analysis due to insufficient number of data points.

**Figure 5:**
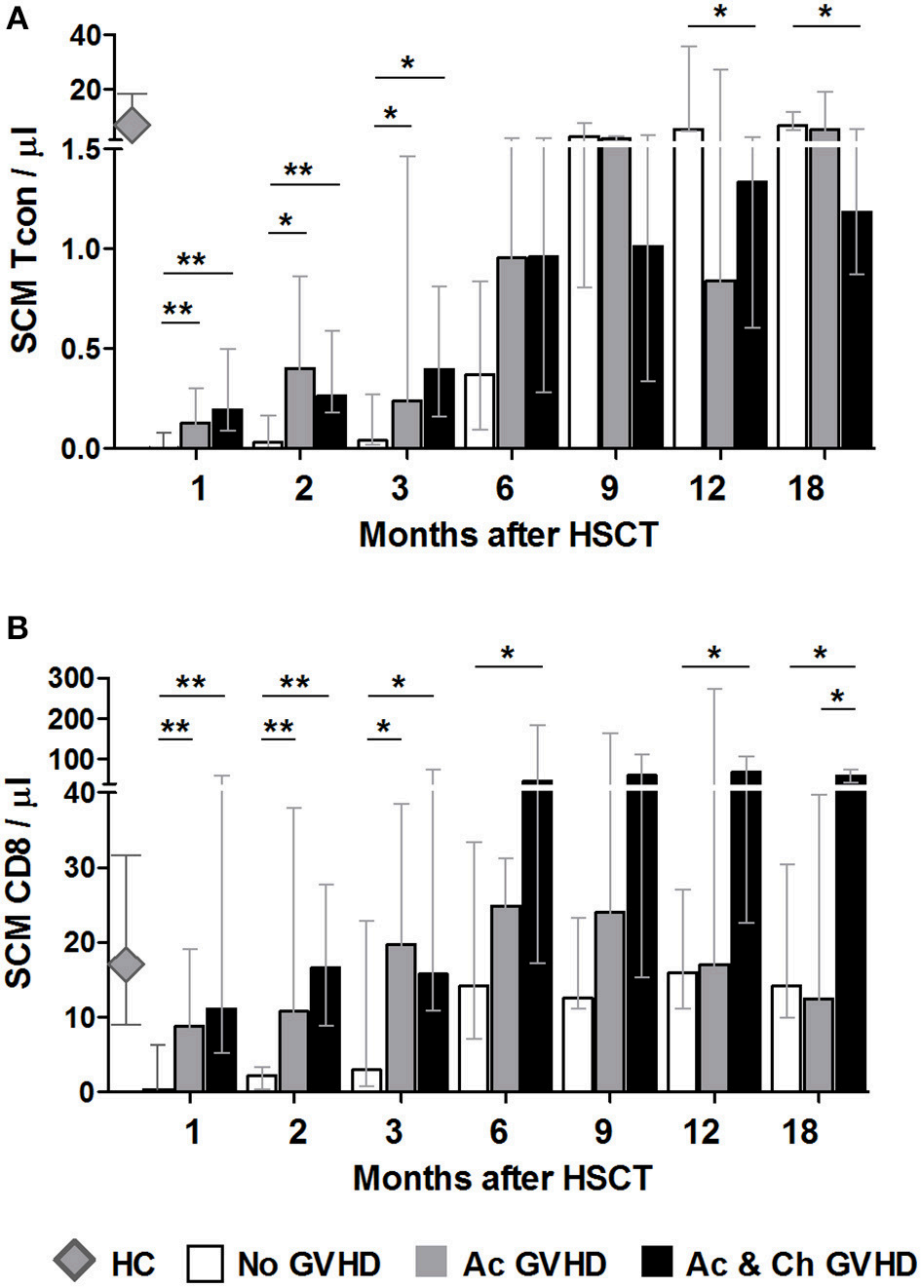
The effect of acute GVHD on SCM Tcon and CD8 reconstitution. SCM absolute counts for Tcon **(A)** and CD8 **(B)** in patients without any form of GVHD (No GVHD, white bars), patients with acute GVHD only (Ac GVHD, gray bars), patients with acute and chronic GVHD (Ac & Ch GVHD, black bars) and healthy controls (HC, gray diamonds). Bar graphs shows median values and IQR. Asterisks denote statistically significant differences between patient groups (^*^p = 0.01 to 0.05; ^**^p = 0.001 to 0.01).

Stem Cell Memory (SCM) Tcon numbers were significantly increased during the first 3 months in GVHD vs. No GVHD patients (Figure 5A). At later time-points, this pattern was reversed and while SCM Tcon reconstituted in the No GVHD and Ac GVHD groups, they became significantly reduced in the Ac & Ch GVHD patient group. A similar pattern was observed when analyzing SCM percentages within Tcon (data not shown). Overall, these data suggest that GVHD development is associated to early increases in SCM Tcon.

For CD8 T cells, initial time-points showed a similar reconstitution pattern to Tcon, whereby patients with GVHD showed significantly increased SCM as compared to No GVHD (Figure 5B). Interestingly, from month 6 onwards patients with aGVHD showed similar SCM CD8 counts as patients who did not develop any form of GVHD. On the other hand, patients who subsequently develop cGVHD appear to sustain a significant increase in this population throughout the follow-up period when compared to the other two groups of patients. A similar pattern was observed when analyzing SCM percentages within CD8 (data not shown).

These data suggest that GVHD development is associated to early increase in the SCM subset after transplant. Furthermore, we observed a sustained increase in this CD8 subset in patients who subsequently develop cGVHD, further pointing to a possible role for these cells in disease development.

### Chronic GVHD Is Associated With Reduced CD4 TCR Vβ Diversity

**Figure 6:**
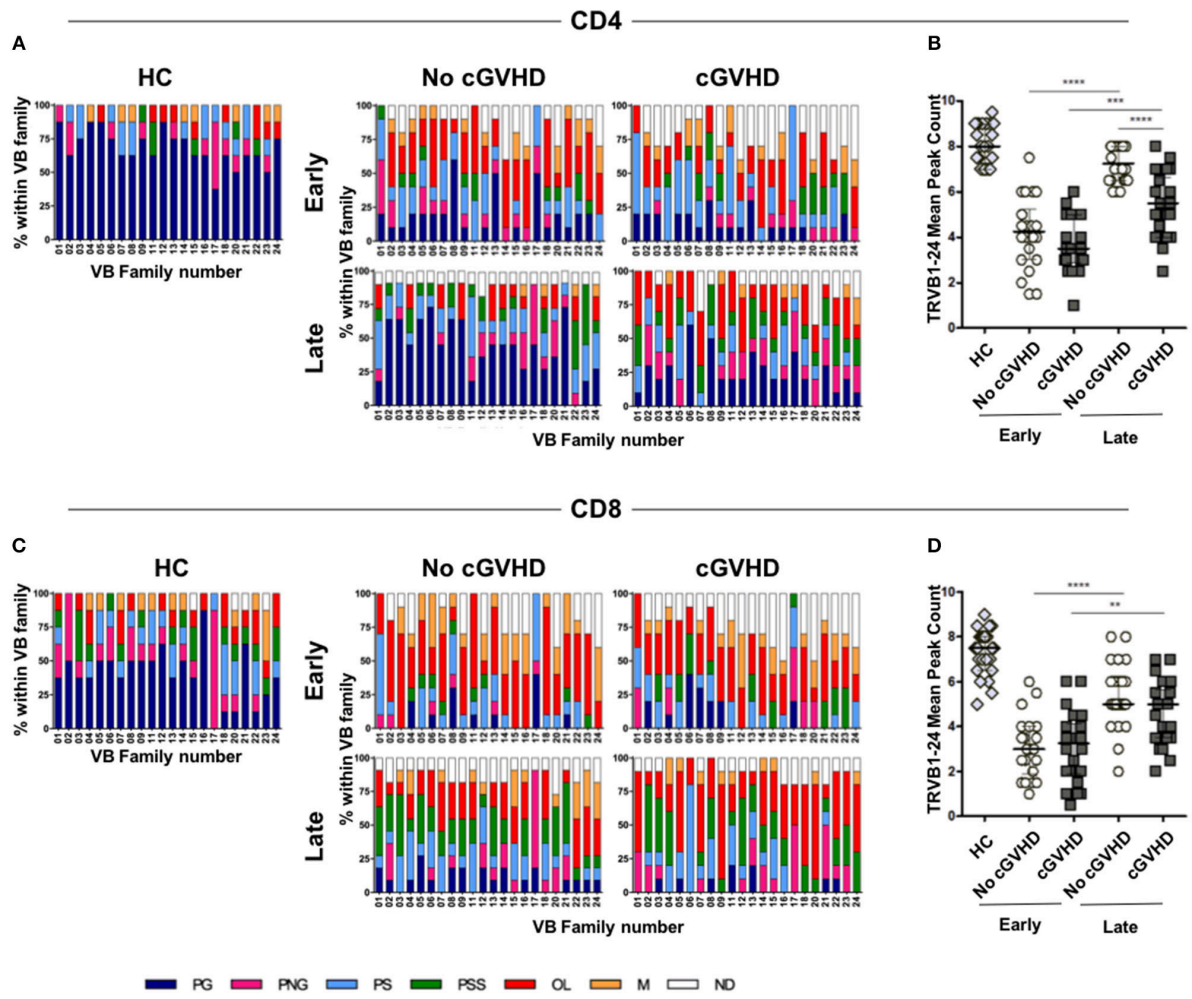
TCR repertoire analysis in HSCT patients and controls. TCRVB diversity analysis was performed on purified CD4 and CD8 T cells from healthy controls (HC) and patients with (cGVHD) and without cGVHD (No cGVHD). Data shown represent values obtained from patient samples early (month 3) and late (months 9 or 12) after HSCT. Clonality analysis of TRB locus transcripts is displayed as profile frequencies of CDR3 spectratypes for TRVB1 to TRVB24 gene families. Profiles were classified on the basis of peak number, distribution shape and relative fluorescence intensity (RFI) as: polyclonal Gaussian (PG, ≥8 peaks with a Gaussian distribution), polyclonal non-Gaussian (PNG, ≥8 peaks with an uneven distribution), polyclonal skewed (PS, 6 peaks or 1 peak with RFI>40%), polyclonal severely skewed (PSS, 1 or 2 peaks with RFI>70%), oligoclonal (O, 2 to 3 peaks), monoclonal (M, 1 peak or a peak with RFI>90%), and not detected (ND). Color stacked columns represent the mean frequency for each of the clonality profiles in CD4 **(A)** and CD8 **(C)** in HC, as well as in patients with and without cGVHD early and late after HSCT. TRVB1-24 CDR3 median peak count in CD4 **(B)** and CD8 **(D)** T cells for HC and patient groups. Asterisks denote statistically significant differences between patient groups (^**^p = 0.001 to 0.01; ^***^p = 0.0001 to 0.001;^****^p < 0.0001).

TCR repertoire diversity was analyzed in purified CD4 and CD8 T cells from patients at early (month 3) and late time-points (months 9 or 12) post-HSCT by TCRVB spectratyping (Figure 6).

Early post-HSCT, we observed no significant differences in TCR diversity between patient groups in either CD4 or CD8 cells (Figures 6B,D). There were few VB families displaying polyclonal distributions, while those with skewed, oligoclonal, and monoclonal distributions prevailed (Figures 6A,C). When early and late time-points were compared within each patient group, significant increases in TCR diversity were observed, suggesting *de novo* CD4 and CD8 T cell production during patient follow-up.

However, when patients developing cGVHD were compared to No cGVHD, a significant reduction in CD4 TCR diversity in cGVHD was observed at later time-points (Figure 6B). This was reflected in decreased prevalence of polyclonal Gaussian profiles and the appearance of numerous skewed profiles (Figure 6A), translating into decreased TCRVB peaks within CD4 T cells in cGVHD (Figure 6B).

Similarly to CD4, we observed an increase in TCR diversity from early to late time-points in CD8. However, the median number of CDR3 peak count within the VB families did not show statistically significant differences between patient groups (Figure 6D). This was likely the result of the observed high prevalence of skewed distributions in CD8 T cells from both patient groups (Figure 6C).

### Chronic GVHD Negatively Impacts Thymic Function

**Figure 7:**
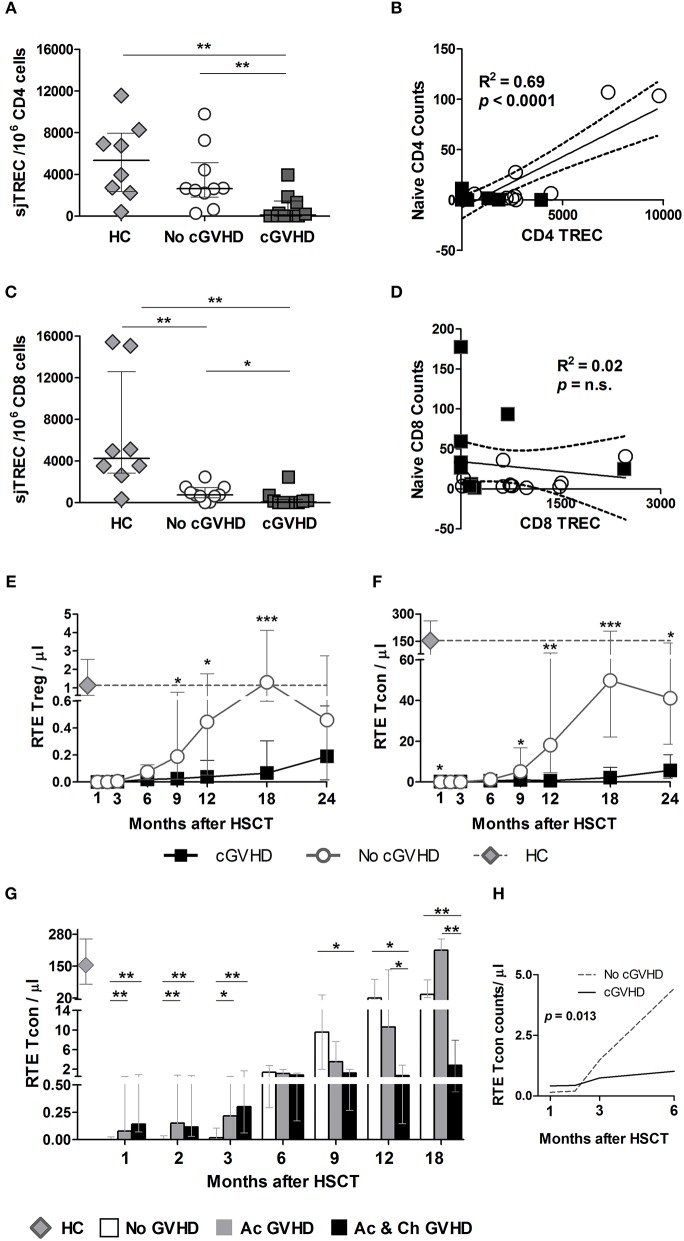
sjTREC content and RTE analysis post-HSCT. sjTRECs were quantified in purified CD4 **(A)** and CD8 **(C)** T cells at months 9 and 12 post-HSCT. Median and IQR values for sjTREC content are shown per 10^6^ CD8 or CD4 T cells in healthy controls (HC, gray diamonds), patients without cGVHD (No cGVHD, white circles), and patients with (cGVHD, dark gray squares). Linear regression analysis between Naive CD4 **(B)** or CD8 **(D)** absolute counts and sjTREC content where black squares represent cGVHD and while circles No cGVHD patients. Absolute counts of RTE Treg **(E)** and RTE Tcon **(F)** in patients with (black lines and squares) and without (gray lines and circles) cGVHD, as well as in HC (gray diamonds and gray dotted lines). **(G)** RTE Tcon counts in patients without GVHD (No GVHD, white bars), with acute GVHD only (Ac GVHD, gray bars), with acute and chronic GVHD (Ac & Ch GVHD, black bars), and in HC (gray diamond). **(H)** RTE Tcon counts on the first 6 months after HSCT in patients with (cGVHD, black line) and without (No cGVHD, dotted gray line) cGVHD, illustrating the results of the LME for these subsets. Median and IQR values are shown. Asterisks denote statistically significant differences between patient groups (^*^p = 0.01 to 0.05; ^**^p = 0.001 to 0.01; ^***^p = 0.0001 to 0.001).

We next analyzed sjTREC content as an estimate of thymic activity in purified CD4 and CD8 T cells at early (month 3) and late (months 9 or 12) time-points post-HSCT. Unfortunately, insufficient cell numbers precluded the analysis at month 3. sjTRECs were significantly reduced in CD4 (Figure 7A) and CD8 (Figure 7C) T cells in cGVHD vs. No cGVHD patients at late time-points.

Importantly, sjTREC content and Naive counts from all patients were positively correlated in CD4 (Figure 7B) but not in CD8 T cells (Figure 7D). Furthermore, Naive CD4 counts were positively correlated with sjTRECs in No cGVHD, but not in cGVHD (data not shown). In CD8 T cells, no significant correlation was observed between sjTREC content and Naive CD8 counts in either patient group (data not shown).

The presence of a significant correlation between CD4 TREC numbers and Naive CD4 counts at month 9 suggests that the Naive CD4 T cell reconstitution observed in patients who do not develop cGVHD is likely the result of *de novo* thymic production. On the other hand, the increase in Naive CD8 T cells observed in patients with cGVHD may depend more on T cell peripheral expansion.

### Recent Thymic Emigrant (RTE) Tcon and Treg in GVHD

The expression of CD31 within Naive CD4 T cells has been reported to contain a population enriched in RTE (26). We therefore quantified this population in our patient cohort as an additional measure of thymic function. We found that patients developing cGVHD had significantly reduced RTE Treg (Figure 7E) and Tcon (Figure 7F) from month 9 onwards. Furthermore, LME analysis of the first 6 months revealed significantly different slopes for RTE Tcon absolute counts (Figure 7H) and percentages (*p* = 0.0032) (data not shown), whereby RTE Tcon increase in the absence of cGVHD with negligible recovery of this population in patients developing cGVHD.

The addition of aGVHD as a variable to the LME analysis of RTE Treg and Tcon showed that aGVHD was associated with increased RTE Tcon counts and percentages during the first 6 months post-HSCT (*p* < 0.01). No effect was observed at later time-points. Accordingly, when the No cGVHD patient group was split into No GVHD and aGVHD only as described earlier, RTE Tcon were significantly decreased in No GVHD vs. both GVHD patient groups at months 1, 2, and 3 (Figure 7G). After month 6, RTE Tcon counts increased in No GVHD and Ac GVHD, while Ac & Ch GVHD patients showed significantly reduced RTE Tcon numbers, suggesting that cGVHD may impact on RTE Tcon reconstitution.

## Discussion

Chronic GVHD remains a major hurdle in allo-HSCT. The significant role played by T lymphocytes in cGVHD pathophysiology has been highlighted in studies that identify graft T cells as major mediators in GVHD development, with Naive T cells playing a central role (27). This has led to the development of T cell depletion strategies that, despite reducing GVHD incidence, are associated with delayed immune reconstitution and increased relapse (28). In order to further study the role played by naive and memory T cell subsets in cGVHD in a setting of partial *in vivo* T cell depletion, we studied a homogenous cohort of patients undergoing unrelated donor allo-HSCT up to 24 months after transplant. This patient cohort was transplanted at a single institution, received the same ATG-containing RIC and GVHD prophylaxis regimens. Hence, despite the negative effects of some immunosuppressive drugs such as CsA, which negatively impacts on Treg function (29), all patients were under the same regimen during the initial post-transplantation period. After 6–9 Months post-HSCT, CsA was discontinued in patients who did not present cGVHD and therefore findings at these later time points may relate to both cGVHD development and/or the immunosuppressive regimen. In this setting, we observed 45% cGVHD incidence (27.5% moderate and severe), likely associated to the fact that 13 out of the 18 patients received PBSC as the graft source and that the majority of these patients had some degree of HLA mismatch with their donors.

While most reports focus on the effects of ATG on cGVHD incidence and immune reconstitution, we investigated the association between naive and memory T cell homeostasis and cGVHD after an ATG-containing conditioning regimen, which is understudied. All our patients received thymoglobulin in the conditioning regimen, which is one of the available rabbit ATGs. Since all of these lymphocyte depleting products are different, it is possible that immune reconstitution in patients receiving another ATG could be somewhat different.

We performed a phenotypic study of T cell reconstitution evaluating Naive, CM, EM, and EMRA reconstitution at fixed time-points after HSCT. We further extend previous studies looking at T cell subsets in cGVHD by investigating the reconstitution of a recently described T cell subset with unique characteristics. SCM have been described as a subset of memory T cells with a Naive-like phenotype, that includes CD45RA, CD62L, and CCR7, that can be distinguished from Naive T cells by the expression of CD95 (30). SCM T cells originate from *in vivo* priming of Naive T cells and possess stem-cell-like properties, being able to generate other memory subsets (25, 30, 31), and are therefore thought to play a role in the maintenance of long term memory. In addition, increased SCM have been associated to autoimmune conditions (32, 33). In order to study the mechanisms involved in T cell reconstitution, we further evaluated proliferation through Ki-67 expression, susceptibility to apoptosis through Bcl-2 and CD95 levels, and evaluated thymic production of CD4 T cells through the identification of CD31-expressing RTE. At selected time-points, we performed TCR diversity and TREC content analysis. Despite the inherent variability of studies performed in human subjects with a distinct genetic makeup, we report interesting observations that shed some light into the biology of cGVHD in humans.

We observed altered Treg homeostasis in patients developing cGVHD. This is consistent with previous reports from our group and others, in the setting of multiple conditioning and GVHD prophylaxis regimens, showing impaired Treg reconstitution in cGVHD (34–37). Reduced Treg in cGVHD was not associated with altered expression of the apoptosis-related proteins Bcl-2 and CD95, but correlated with decreased proliferation, possibly associated with immunosuppressive therapies. Importantly, we demonstrate a clear association between cGVHD development and severely impaired Naive and SCM Treg reconstitution in this setting. Decreased Naive Treg may result from reduced thymic output following GVHD-induced thymic damage. Indeed, we found significantly decreased levels of the RTE-enriched CD45RA^+^CD62L^+^CD31^+^ subset, a population that has been used as an indicator of thymic production, within Treg in cGVHD (26, 38). Decreased SCM Treg may result from decreased Naive Treg and/or differentiation into memory phenotypes.

Overall, our data on Treg subset reconstitution support the hypothesis that imbalances in T cell tolerance play an important role in the biology of cGVHD and reinforces the potential benefits of Treg-restoring therapies, particularly of Naive Treg as this subset is particularly depleted in cGVHD.

In line with the previously reported negative impact of ATG on CD4 T cell reconstitution (39–41), Tcon recovery was impaired in both patient groups. However, we did not find significant differences in total Tcon numbers when comparing patients with and without cGVHD. Nevertheless, we report significantly distinct Tcon subset composition when comparing both patient groups. Notably, Naive and SCM Tcon recovery were impaired in patients developing cGVHD. This was accompanied by a tendency for increased levels of the more differentiated subsets EM and EMRA in patients with cGVHD

T cell reconstitution is thought to occur through peripheral expansion and *de novo* thymic production (42, 43). In order to investigate the pathways leading to impaired Naive Tcon reconstitution, we measured *ex vivo* proliferation throughout the follow-up, using Ki-67 as a proliferation marker. We observed a tendency for reduced Ki-67 expression within total Tcon, as well as in Tcon subsets (data not shown), in patients developing cGVHD. Susceptibility to apoptosis, as assessed by the quantification of Bcl-2 and CD95 expression levels, was similar between patient groups, pointing to decreased homeostatic proliferation as a contributing factor to impaired Naive Tcon reconstitution.

In order to have a measure for *de novo* thymic production, we quantified sjTREC in total CD4 T cells. TRECs are episomal DNA sequences formed during thymic T-cell development by TCR V-J gene rearrangements that correlate with thymopoiesis, particularly in the absence of extensive proliferation (44). sjTRECs were significantly reduced in CD4 T cells from cGVHD *versus* No cGVHD patients. Interestingly, CD4 sjTREC correlated with Naive CD4 numbers, suggesting that, in the absence of cGVHD, the Naive Treg and Tcon recovery is likely to result from *de novo* thymic production occurring after HSCT (45). In order to further detail the contribution of thymic output to CD4 T cell recovery post-transplant, we quantified RTE Tcon. We show that from month 9 onwards, RTE Tcon increased in the absence of cGVHD while patients who develop cGVHD show a severely impaired RTE reconstitution. It is therefore likely that a combination of decreased proliferation and impaired thymic output leads to impaired Naive Tcon and Treg reconstitution in cGVHD. In addition, CD4 TCRVB diversity was significantly reduced in patients, more so in cGVHD, further pointing to impaired thymic production in patients developing cGVHD.

When accessing the role played by acute GVHD in RTE Tcon recovery after HSCT, we found increased RTE Tcon at months 1–3, both in Acute GVHD and in Acute and Chronic GVHD patients, as compared to No GVHD. This suggests a potential deleterious role of RTE Tcon early after HSCT. It is unclear if these cells are of thymic origin or result from cytokine-driven proliferation of infused RTE (46). We speculate that RTE Tcon detected early after HSCT may contain self-reactive clones, while RTE Tcon present at later time-points in No cGVHD result from adequate thymic selection processes.

Overall, we observed impaired Naive and SCM Tcon reconstitution in cGVHD, associated with reduced peripheral expansion. In addition, decreased TREC content and RTE Tcon recovery further suggest that cGVHD results from alterations in *de novo* thymic production. Intriguingly, we found that cGVHD development was associated to increased RTE Tcon early after HSCT through yet undescribed mechanisms.

It is noteworthy that we report a distinct picture emerging for CD8 T cells. EM, EMRA and CM were the most abundant CD8 subsets in the post-transplantation period. Interestingly, cGVHD associated with increased Naive, SCM and EMRA as compared to patients who did not develop cGVHD.

Stem Cell Memory (SCM) have been reported to differentiate from Naive, to be increased in autoimmune disease (32, 33) and to induce lethal GVHD in mouse studies (47). Our findings of increased Naive and SCM CD8 in cGVHD vs. No cGVHD raise the possibility that Naive CD8 T cells may differentiate into SCM and be involved in GVHD development by yet undescribed mechanisms. Interestingly, when the effects of previous aGVHD were taken into consideration in our statistical analysis, we observed that during the first 6 months after transplant SCM Tcon and CD8 were significantly affected by aGVHD. The stratification of patient groups into No GVHD, Acute GVHD only and Acute & Chronic GVHD confirmed that during these initial stages after HCST patients developing GVHD show increased numbers of these cells. A sustained increase in this population in patients developing cGVHD was observed for CD8 but not Tcon, further suggesting a potential role for CD8 SCM is cGVHD development.

Other studies have shown that the thymus is a direct target of GVHD (7) resulting on the emergence of self-reactive clones and the autoimmunity associated to cGVHD (48, 49). We observed a high prevalence of skewed and oligoclonal TCR repertoires in CD8 from both patient groups. Interestingly, the significantly reduced CD8 sjTREC content in cGVHD vs. No cGVHD patients suggests reduced thymic output and/or a high level of homeostatic proliferation observed in these patients.

Our data raises the possibility that increased Naive and SCM CD8 present from early time-points after transplant in cGVHD patients, may originate from defective negative selection mechanisms resulting from thymic tissue damage, leading to the output of self-reactive CD8 clones that may further differentiate and mediate disease. Given the increased levels of homeostatic T cell proliferation in the post-transplantation period, we cannot exclude the possible contribution of cell proliferation to the observed increase in Naive and SCM CD8.

We therefore extend on the observations by Alho et al. (34) that reported increased Naive T cell subsets in patients developing cGVHD, by comparing patients with and without cGVHD at month 3 after HSCT (34). We now show that this occurs not only within Naive CD8 but also in the SCM CD8 T cell subset, by detailing increases in these cell populations in patients who develop cGVHD in a cohort of patients undergoing unrelated HSCT after an ATG-containing conditioning regimen. We further show that this occurs in CD8 T cells in a sustained manner from month 1 to 9 in both percentages and absolute counts. In addition, by stratifying our patient cohort with regards to previous history of acute GVHD, we observed that increased SCM in Tcon and CD8 occurs early after HSCT in patients developing aGVHD. Of note, we did not observe any statistically significant impact of adding aGVHD as a co-variate within the Treg subsets.

In summary, we show that after an ATG-containing RIC regimen, cGVHD development after allo-HSCT is associated with reduced Treg recovery, particularly of Naive and SCM subsets. We speculate that this is likely due to reduced thymic production within the CD4 T cell compartment, where significantly decreased TCRVB diversity is observed in patients developing cGVHD. We also show that cGVHD development is associated to increases in Naive, SCM and EMRA CD8 T cells. This becomes apparent early after HSCT and persists throughout our 24-month follow-up, suggesting a potential involvement of these cells in the development of cGVHD.

## Ethics Statement

This study was carried out in accordance with the recommendations of the Ethics Committee of Lisbon Academic Medical Center with written informed consent from all subjects. All subject gave written informed consent in accordance with the Declaration of Helsinki. The protocol was approved by Ethics Committee of Lisbon Academic Medical Center (ref. 459/13).

## Author Contributions

MS analyzed data and wrote the paper. RA designed experiments, performed experiments, analyzed data, and wrote paper. IF, SB, and AR performed experiments. AV performed cell sorting and flow cytometry support. PP and NC collected clinical data. DL performed the spectratyping and TREC analysis. RR performed statistical data analysis. AA and AS analyzed data. CM, FL, and RM recruited patients. JR and JL conceived and designed the study. JL recruited patients, designed experiments, and wrote the paper.

### Conflict of Interest Statement

The authors declare that the research was conducted in the absence of any commercial or financial relationships that could be construed as a potential conflict of interest.

## Abbreviations

aGVHD: acute GVHD
ALL: acute lymphoid leukemia
AML: acute myeloid leukemia
CLL: chronic lymphocytic leukemia
CML: chronic myeloid leukemia
cGVHD: chronic GVHD
CMV: cytomegalovirus
F: female
G: grade
GVHD: Graft versus Host Disease
HL: Hodgkin lymphoma
HLA: human leukocyte antigen
M: male
MDS: myelodysplastic syndrome
MF: Myelofibrosis
MM: multiple myeloma
NHL: non-Hodgkin lymphoma
PBSC: peripheral blood stem cell.
