# Supplementary material for: Naive and Stem Cell Memory T Cell Subset Recovery Reveals Opposing Reconstitution Patterns in CD4 and CD8 T Cells in Chronic Graft vs. Host Disease
Source: Front Immunol. 2019 Mar 6;10:334. doi: 10.3389/fimmu.2019.00334 (PMC6414429; doi:10.3389/fimmu.2019.00334)
Supplement: Supplementary file 1 [file Data_Sheet_1.PDF]

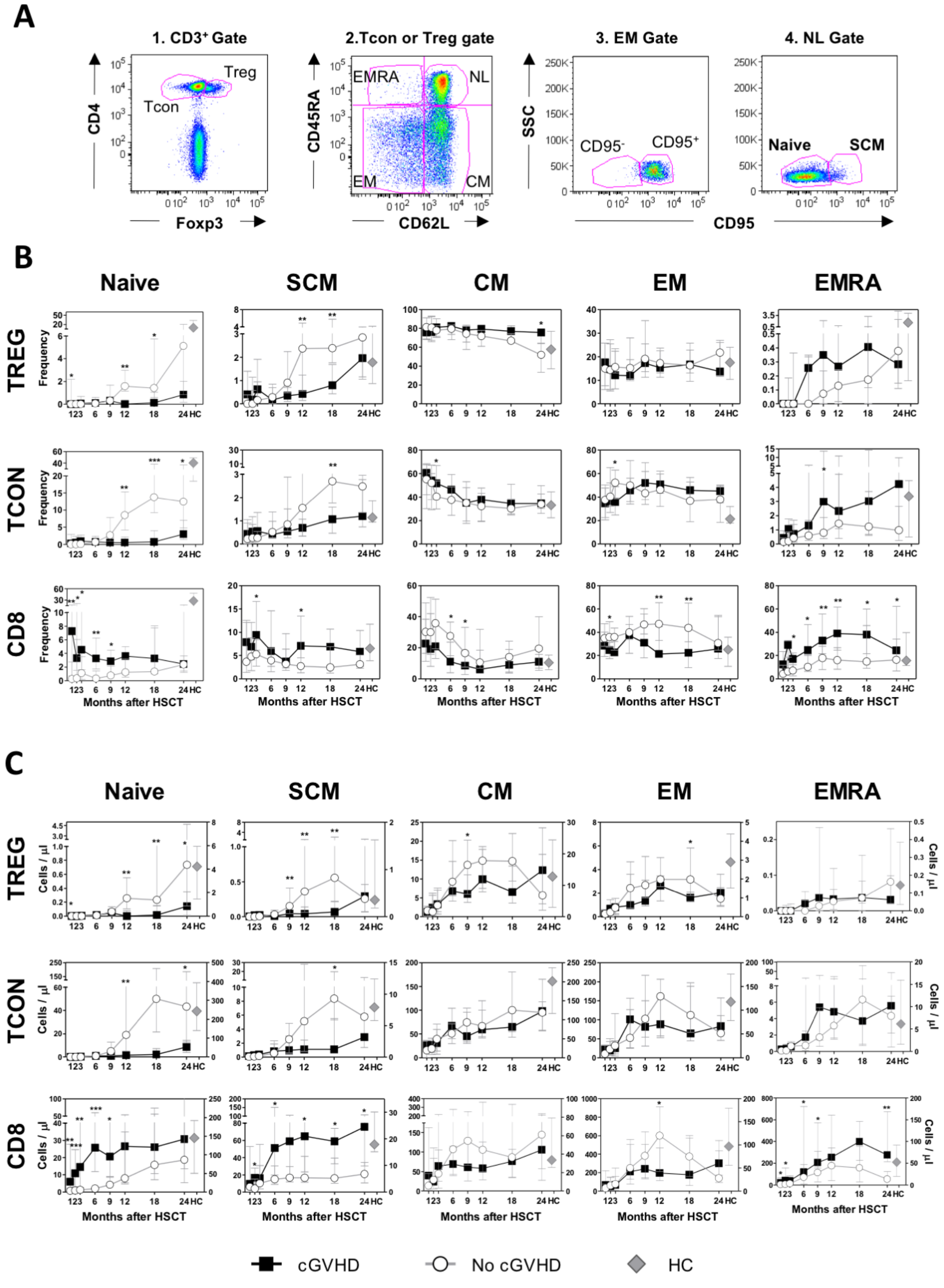

Supplemental Figure 1. Treg, Tcon and CD8 subset reconstitution in cGVHD and No cGVHD patients **(A)** Flow cytometry plots illustrating the gating strategy used to identify Naive and Memory subsets. Treg were identified as CD4+ Foxp3+, Tcon as CD4+ Foxp3- and CD8 as CD4-, all within CD3+ lymphocytes. T cell populations were further divided into subsets according to CD45RA and CD62L expression: Central memory (CM, CD45RA- CD62L+), Effector Memory (EM, CD45RA- CD62L-), CD45RA+ Effector Memory (EMRA, CD45RA+ CD62L-), Naive–like (NL, CD45RA+CD62L+) cells. Stem Cell Memory (SCM) as CD45RA+ CD62L+CD95+ and Naive as CD45RA+ CD62L+CD95- **(B)** Median percentages for each subset within Treg, Tcon and CD8 cells. **(C)** Median absolute cell number per µl for Treg, Tcon and CD8 subsets. No cGVHD: grey lines and circles; with cGVHD: black lines and squares; HC: grey diamonds and dotted lines. Median counts from patients are shown on the left and HC on the right y axis. Symbols represent median values ± IQR. (*p=0.01 to 0.05; **p=0.001 to 0.01).
